# Post-stroke inflammation and the potential efficacy of novel stem cell therapies: focus on amnion epithelial cells

**DOI:** 10.3389/fncel.2012.00066

**Published:** 2013-01-17

**Authors:** Brad R. S. Broughton, Rebecca Lim, Thiruma V. Arumugam, Grant R. Drummond, Euan M. Wallace, Christopher G. Sobey

**Affiliations:** ^1^Vascular Biology and Immunopharmacology Group, Department of Pharmacology, Monash UniversityClayton, VIC, Australia; ^2^The Ritchie Centre, Monash Institute of Medical ResearchClayton, VIC, Australia; ^3^School of Biomedical Sciences, University of QueenslandBrisbane, QLD, Australia

**Keywords:** stem cells, human amnion epithelial cells, stroke, immune cells, inflammation, cerebral ischemia, therapy

## Abstract

Ischemic stroke is a debilitating disease for which there are currently no effective treatments besides the clot-buster, tissue plasminogen activator (t-PA), which is administered to less than 10% of patients due to a limited (4.5 h) time window of efficacy. Thus, there is an urgent need for novel therapies that can prevent or reverse the effects of stroke-induced brain injury. Recent encouraging reports have revealed that stem cells derived from human tissue, including embryonic, induced pluripotent, neural, and mesenchymal cells, can rescue injured brain tissue and improve functional recovery in experimental models of stroke. However, there are potentially major limitations to each of these types of stem cells that may ultimately prevent or restrict their use as viable mainstream treatment options for stroke patients. Conversely, stem cells derived from the placenta, such as human amnion epithelial cells (hAECs), appear to have several important advantages over other stem cell lineages, in particular their non-tumorigenic and non-immunogenic characteristics. Surprisingly, so far hAECs have received little attention as a potential stroke therapy. This brief review will firstly describe the inflammatory response and immune cell involvement following stroke, and then consider the potential for hAECs to improve stroke outcome given their unique characteristics. These actions of hAECs may involve a reduction of local inflammation and modulation of the immune response, promotion of neural recovery, differentiation into neural tissue, re-innervation of lost connections, and secretion of necessary cytokines, growth factors, hormones and/or neurotransmitters to restore cellular function.

## Introduction

Stroke occurs following a sudden disruption of blood flow to the brain, thereby starving the tissue of oxygen and nutrients and initiating neuronal death within minutes (Broughton et al., 2009). This crippling disease is the world's second leading cause of death, with approximately 15 million strokes occurring each year, and accounting for 9.5% (i.e., 6 million) of all deaths per annum (WHO, 2002). Alarmingly, the incidence of stroke more than doubles each successive decade for people over the age of 55. Thus, with the annual number of strokes increasing due to the ageing population, an increasingly greater financial and social burden will be caused to survivors and to the community. Thus, major advances to prevent and treat stroke are of paramount importance.

### Treatment of ischemic stroke

Ischemic stroke, which occurs when the blood supply to the brain is obstructed by an embolus or a thrombus, accounts for approximately 87% of all stroke cases (WHO, 2002). It is most disappointing that still the only available “pharmacological” intervention to reduce brain damage after stroke is the “clot-buster,” tissue plasminogen activator (t-PA). t-PA is an enzyme that works by catalyzing the conversion of plasminogen to plasmin which can then break down either the embolus or thrombus causing cerebral ischemia (Sloan, 1987). However, t-PA can only be administered within 4.5 h of the onset of ischemia and only after a CT scan has verified that the stroke is due to a thrombus rather than a hemorrhage (Del Zoppo et al., 2009). After 4.5 h, there is no evidence for a net beneficial effect of t-PA due to the increased risk of hemorrhagic transformation. Consequently, only 2–8% of stroke patients currently receive this treatment (based on United States statistics) (Reeves et al., 2005; Kleindorfer et al., 2008). At best, t-PA can only restore blood flow, and it cannot target mechanisms of cellular injury or others that promote healing. Other treatment options for ischemic stroke include anti-coagulants, such as heparin that inhibits clot formation, and anti-platelet agents, such as aspirin, that reduce the risk of platelet aggregation. However, these treatments have no effect on stroke outcome and are mainly used in the prevention of a secondary stroke. Although a plethora of neuroprotective compounds have shown promise in animal models of stroke, no other treatment has achieved efficacy in clinical trials (Dirnagl, 2006). Therefore, new therapies that may protect neural tissue from post-ischemic damage and/or promote functional recovery are desperately needed to reduce mortality and long-term neurological deficits in stroke victims. An important step in this process will be to understand the key mechanisms that contribute to injury following stroke.

### Activation of the immune system and brain inflammation after stroke

**Figure 1:**
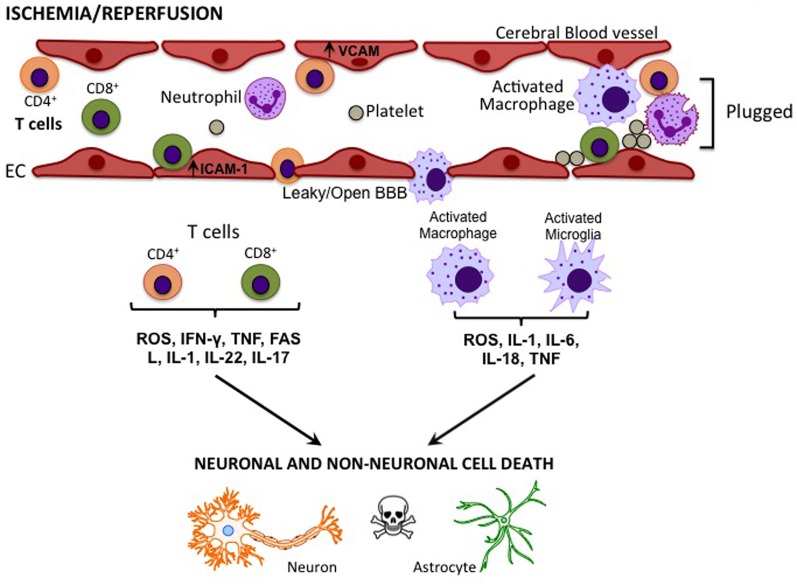
**Cytokines/effector molecules involved in post-stroke inflammation in the brain.** Following cerebral ischemia-reperfusion, elevated levels of adhesion molecules, such as ICAM-1 and VCAM in vascular endothelial cells (EC), attract circulating T cells and macrophages. These immune cells may cross the compromised blood-brain barrier (BBB) to infiltrate the injured tissue and cause cell death via the release of various cytokines and effector molecules.

It is now understood that the immune system plays an integral role in the pathogenesis of ischemic stroke and it contributes to infarct formation (Iadecola and Anrather, 2011). In the post-stroke acute phase (minutes to hours), microglia and cerebral endothelial cells within the affected zone are activated by hypoxia, shear stress, and the production of reactive oxygen species (ROS) (Jin et al., 2010). This causes the expression of adhesion molecules such as intercellular adhesion molecule-1 (ICAM-1), vascular adhesion molecules (VCAMs), selectins (in particular, P-selectin and E-selectin), and integrins (in particular, Mac-1 and LFA-1), on endothelial cells, leukocytes, and platelets (Yilmaz and Granger, 2010). Adhesion molecule expression is also induced on circulating leukocytes. Simultaneously, oxidative stress and locally-derived pro-inflammatory mediators (cytokines and chemokines) produced by the injured tissue alter the permeability of the blood brain barrier (BBB). As the ischemic cascade progresses, cell death leads to a new phase of the inflammatory response. Dying and dead cells release “danger signals” that activate the immune system (Iadecola and Anrather, 2011; Magnus et al., 2012). Some of these signals, such as the nucleotides adenosine triphosphate (ATP) and uridine triphosphate (UTP) and high-mobility group protein B1 (HMGB1), are released by cells under stress when the cell membrane is still intact, and thereby set the stage for the subsequent immune response. A result of these processes is a time-dependent infiltration of immune cells (Figure 1). These immune cells include neutrophils, macrophages, dendritic cells, and T and B lymphocytes (Stevens et al., 2002; Gelderblom et al., 2009, 2012; Brait et al., 2010; Jin et al., 2010; Kleinschnitz et al., 2010, 2012). Neutrophils, which are thought to be the first immune cell to enter the brain post-stroke, undergo granule exocytosis to release a variety of pro-inflammatory molecules such as large quantities of nitric oxide (NO) derived from inducible NO synthase, nicotinamide adenine dinucleotide phosphate (NADPH) oxidase-derived ROS, and matrix metalloproteinases (MMPs) (Yilmaz and Granger, 2010). Both CD4^+^ and CD8^+^ T lymphocytes contribute to brain injury by producing pro-inflammatory mediators, such as the potent cytokines interferon-γ (IFN-γ), interleukin-6 (IL-6), IL-17, and tumor necrosis factor (TNF) (Kleinschnitz et al., 2010, 2012; Brait et al., 2012; Gelderblom et al., 2012). T lymphocytes contribute further to the state of oxidative stress by also producing NADPH oxidase-derived superoxide (Brait et al., 2010). In addition, CD8^+^ T lymphocytes induce apoptosis in already compromised neuronal cells following stroke (Barry and Bleackley, 2002; Brait et al., 2012), and both macrophages and activated microglia produce a number of pro-inflammatory cytokines and ROS to exert neurotoxic effects (Jin et al., 2010).

Interestingly, the spleen appears to play an important role in the acute influx of immune cells into the brain after stroke. For example, splenectomy prior to experimental stroke reduces infarct size (Ajmo et al., 2008). Moreover, these authors showed that splenectomy-induced neuroprotection following stroke correlated with a decrease in activated microglia, macrophages, and neutrophils in the ischemic hemisphere, thus suggesting that the spleen is a possible source of detrimental immune cells following stroke (Ajmo et al., 2008). In addition, Offner et al. found that T lymphocytes derived from blood and lymph nodes secreted increased levels of pro-inflammatory mediators, and expressed elevated levels of chemokine receptors post-stroke (Offner et al., 2006). More recently, it was shown that the spleen contributes to neurodegeneration following stroke through IFN-γ signaling (Seifert et al., 2012).

### The systemic immune response following stroke

The immune response following stroke is not restricted to the brain, as effects on immune function are also seen in the periphery. While both innate and adaptive immune cells contribute to early post-stroke neuronal injury, the circulating levels of these cells are then rapidly reduced. For example a profound systemic immunodepression—or “stroke-induced immunodeficiency syndrome”—occurs as early as 12 h after ischemic stroke (Gendron et al., 2002; Prass et al., 2003). This phenomenon is triggered by hyperactivity of the sympathetic nervous system and the hypothalamic–pituitary–adrenal axis due to post-stroke brain damage, which leads to reduced numbers of T and B lymphocytes and also of NK cells within the spleen, thymus, bone marrow, and lymph nodes (Gendron et al., 2002; Prass et al., 2003; Offner et al., 2006; Liesz et al., 2009). This leads to increased apoptosis and increased release of immune cells from these primary and secondary lymphoid organs, resulting in tissue atrophy and this consequently predisposes patients to infection (e.g., commonly resulting in pneumonia or sepsis), a major determinant of stroke morbidity and mortality.

Interestingly, although the liver is not a secondary lymphoid organ, a recent publication investigated the effect stroke had on invariant natural killer T (iNKT) cells within that organ (Wong et al., 2011). Wong and colleagues showed that cerebral ischemia-reperfusion slowed the migration of resident hepatic iNKT cells and increased the expression of the immunosuppressive cytokine, IL-10, in association with an increased susceptibility to bacterial infection. In support of these findings, the onset of bacterial infection occurred much earlier in iNKT cell-deficient mice subjected to stroke, whereas brain infarct size was unchanged. This indicated that iNKT cells play a key role in systemic protection against infection after stroke. Furthermore, those researchers found that the increased release of noradrenergic neurotransmitters from sympathetic nerves innervating the liver following stroke can undermine systemic immunity by a direct inhibitory effect on hepatic iNKT cells. Thus, the authors suggested that blockade of stress pathways could improve outcomes in stroke patients by helping to protect systemic immune function and thereby preventing infections. Collectively, evidence suggests that various components of the immune system in the brain as well as in secondary lymphoid and visceral organs may play critical roles in the development of post-stroke damage and mortality.

## The potential for stem cell therapy following stroke

Given the complex nature of post-ischemic brain injury and the failure of effective stroke treatments targeting single molecular pathways, ultimately successful approaches may include cell-based therapies that have the potential to target multiple injury mechanisms and cell types when administered at an appropriate time(s) after the stroke event. Hence, there is now considerable interest in stem cell therapy as a possible treatment for stroke. Stem cells are undifferentiated cells capable of self-renewal and are broadly classified as being of embryonic, fetal, or adult origin (Yu et al., 2009).

Embryonic stem cells (ESCs) are pluripotent, meaning that they can give rise to all cell types of the organism, whereas fetal and adult stem cells are multipotent, such that they can give rise to cells of multiple, but limited number of lineages. A variety of stem cells, including embryonic, bone marrow, neural, and induced pluripotent stem cells (iPSCs) have been shown to improve stroke outcome (Daadi et al., 2008; Schwarting et al., 2008; Hicks et al., 2009; Kawai et al., 2010). As a result of such promising studies, the first fully regulated clinical trial (PISCES study—Pilot Investigation of Stem Cells in Stroke) using ESCs to treat stroke patients has recently commenced in Glasgow. Unfortunately however, there tends to be major limitations with the use of most stem cell types, which may offset their use as a clinical treatment for stroke patients.

### Limitations and benefits of stem cell lineages for transplantation into the CNS

ESCs were expected to have broad potential due to their pluripotent capabilities, and transplantation of human ESC neural derivatives into a rodent model of stroke has been reported to improve functional outcome (Daadi et al., 2008; Hicks et al., 2009). Nevertheless, several problems exist regarding human ESCs, including ethical/political issues (i.e., due to the destruction of human embryos), immune rejection, and their fetal “age” (i.e., they lack key functional characteristics of adult cells) (Xi et al., 2010). Moreover, ESCs may form teratomas (developmental tumors) following transplantation (Knoepfler, 2009).

iPSCs were first derived in 2006 (Takahashi and Yamanaka, 2006) by re-programming mouse and human fibroblasts into pluripotent ESC-like cells. Since then, many types of iPSCs have been created using diverse cell types (Kiskinis and Eggan, 2010). iPSCs possess most of the key properties of ESCs but avoid the ethically controversial issues surrounding embryo destruction. These cells have been used to treat central nervous system (CNS) injuries such as spinal cord injury and stroke in rodents (Kawai et al., 2010; Tsuji et al., 2010), but in both cases tumor formation from iPSCs was observed.

Fetal neural stem cells (NSCs) are derived from human fetal brains (isolated from aborted material) and are capable of differentiating into neurons, astrocytes, or oligodendrocytes (Lindvall and Kokaia, 2011). Because of the invasive nature of obtaining autologous adult human neural cells, fetal NSCs have been evaluated as an alternative expandable source of neural cells. Although NSCs also involve ethical issues, these cells were considered to be safer than human ESCs regarding tumor formation after transplantation, however, brain and spinal cord tumors have been reported to develop following fetal NSC treatment (Dirks, 2001; Schmidt et al., 2005; Amariglio et al., 2009).

Mesenchymal stem cells (MSCs), derived from bone marrow or umbilical cord blood, can differentiate into neuronal-like cells, astrocytes, or endothelial cells, and their administration can reduce infarct volume and improve functional outcome in experimental stroke (Wu et al., 2008; Liu et al., 2009). Transplantation of MSCs can also reduce apoptosis and promote endogenous cellular proliferation after stroke, and long-term follow-up data have revealed improved survival in patients that received bone marrow MSCs compared with controls (Lee et al., 2010). Similar to ESCs, however, concerns and limitations associated with MSC use in stroke include poor cell survival and engraftment after transplantation, no direct evidence of functional neuronal differentiation, limited sources, and the fact that their extraction from bone marrow requiring invasive procedures, although they do not appear to form tumors after transplantation (Zimmermann et al., 2003).

### Human amnion epithelial cells

#### hAEC characteristics

While the above stem cell types may certainly have therapeutic potential if their respective issues can be addressed, currently those limitations seriously offset their likely routine use in clinical stroke. An alternative stem cell lineage that is gaining interest as a potential stem cell therapeutic is the human amnion epithelial cell (hAEC). hAECs are derived from the amniotic sac, a thin avascular tissue that encloses the fetus and is attached to the placenta. The amnion consists of an inner layer of epithelial cells that is in direct contact with the amniotic fluid, referred to as the amniotic epithelium. Directly beneath the epithelial layer is the amniotic mesoderm, which includes a compact stromal layer and also a fibroblast layer. These two cell types have a different embryological origin. hAECs are derived from the embryonic ectoderm and amniotic mesodermal cells originate from the embryonic mesoderm (Parolini et al., 2008). Whilst amniotic mesodermal cells are also a potential cell-based therapy for stroke, this review will limit its focus to the potential of hAEC therapy.

A considerable advantage of hAECs over other stem cell lineages is that they possess very few of the limitations of other stem cell types outlined above (see Table 1). hAECs are easily obtained from separating the amnion sac from the term placenta, which are usually discarded after birth (Miki et al., 2005). As such, hAECs are readily available, they require no invasive procedure for harvesting, and they largely lack ethical barriers to their use (Yu et al., 2009). Furthermore, native hAECs do not express the polymorphic antigens HLA-A, HLA-B and HLA-C (class IA), and HLA-DR (class II), on their surfaces (Akle et al., 1981; Terada et al., 2000) but express the non-polymorphic, non-classical human leukocyte antigen G (HLA-G) (Houlihan et al., 1995), which does not elicit an immune response but rather suppresses it. Thus, hAECs are considered to be immunologically inert and would thus be expected to have a very low risk of rejection upon transplantation. These properties are, of course, consistent with the functions of the amnion to protect the fetus from the mother's immune system and to secrete various nutritive factors (Liu et al., 2008). Moreover, hAECs have low tumorigenicity (Miki et al., 2005) because they lack telomerase, an enzyme that preserves chromosomal sequences commonly lost during successive cell division (Hiyama and Hiyama, 2007). It would therefore be expected that hAECs are unlikely to promote tumor formation in the recipient.

**Table 1:** **Beneficial characteristics of five major stem cell lineages**.

**Benefits**	**ESCs**	**BMSCs**	**iPSCs**	**NSCs**	**hAECs**
Readily available	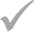	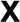	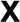	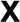	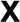
Do not require invasive extraction	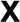	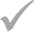	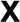	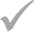	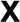
Pluripotent properties	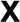	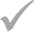	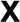	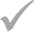	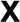
Differentiate into functional neural tissue	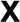	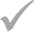	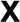	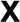	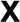
Non-immunogenic	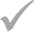	^a^ 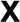	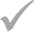	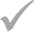	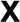
Immunomodulatory properties	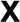	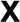	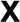	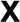	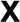
Non-tumorigenic	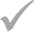	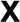	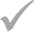	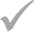	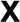

a Autologous transplantation only.

ESCs, embryonic stem cells; BMSCs, bone marrow-derived stem cells; iPSCs, induced-pluripotent stem cells; NSCs, neural stem cells; hAECs, human amnion epithelial cells.

Due to the fact that amnion epithelial cells originate from the epiblast, from which they separate early in embryonic development (day 8), hAECs possess a high level of pluripotency. For example, hAECs can differentiate into all three germ layers: endoderm, ectoderm, and mesoderm (Toda et al., 2007). Notably, they can generate clinically relevant cell types, such as myocytes (including cardiomyocytes), osteocytes, adipocytes, pancreatic cells, hepatocytes, as well as neural, and astrocytic cells (Toda et al., 2007). These latter cell types are, of course, of particular importance for treating stroke. More specifically, hAECs may express markers of glial and neuronal progenitor cells and display multiple neuronal functions, such as synthesis and release of acetylcholine, catecholamines, and neurotrophic factors (Elwan and Sakuragawa, 1997; Bailo et al., 2004). Recent studies have shown that hAECs can facilitate neuroregeneration in CNS disorders such as Parkinson's disease (Kakishita et al., 2000). Thus, there is good reason to predict that hAECs may exert a neuroprotective effect if administered after stroke, but they have so far received little attention as a potential stroke therapy. Before further considering this potential, we will briefly summarise the known effects of hAECs in CNS diseases.

#### hAECs in the treatment of CNS diseases

In animal models of CNS disorders, accumulating evidence suggests that hAECs can exert neuroprotection and facilitate neuroregeneration. For example, hAECs transplanted into the striatum of a rat model of Parkinson's disease were found to not only survive but were functional (i.e., producing dopamine) and prevented neuronal degeneration (Kakishita et al., 2000, 2003). In addition, after injection of hAECs into the transection cavities of a primate model of spinal cord injury, those cells survived for up to 60 days during which there was no evidence of inflammation, suggesting that the cells can avoid immunological rejection within the CNS (Sankar and Muthusamy, 2003). Furthermore, improved performance in locomotor tests was observed in hAEC-treated animals compared to lesion control animals, suggesting neuroprotection and improved function of the motor neuron tracts controlling locomotion. It has also been reported that administration of hAECs assists in the penetration of host axons, and completely abolishes glial scar formation in rats with spinal cord injury (Wu and Hui, 2006). More recently, McDonald et al. reported preliminary findings that intraperitoneal injection of hAECs can suppress clinical symptoms, as well as decrease CNS inflammation, demyelination and axonal degeneration in the spinal cord and brain of a experimental autoimmune encephalomyelitis (EAE) mouse model of multiple sclerosis (McDonald et al., 2011). These authors also found that hAECs can reduce proliferation of myelin oligodendrocyte glycoprotein-specific T cells, and also decrease their secretion of pro-inflammatory cytokines, IFNγ, and TNF. Interestingly, these data point to a possible immunomodulatory mechanism by which amnion epithelial cells can suppress the development of EAE. Consistent with these findings, Liu et al. recently reported that intravenously administered hAECs reduced infiltration of T lymphocytes and monocyte/macrophages, and consequently attenuated demyelination, within the CNS of an EAE mouse (Liu et al., 2012). These authors demonstrated that this effect was due to the secretion of transforming growth factor-β and prostaglandin E2 from hAECs to suppress splenocyte proliferation. As a further example of the beneficial neuroprotective effects of hAECs, conditioned media collected from these cells has been found to be neurotrophic for rat cortical cells (Uchida et al., 2000), and to support the survival of chicken neural retinal cells (Tcheng et al., 1994). Overall, there is mounting evidence that hAECs may have substantial protective and regenerative properties that could be amenable to the treatment of neurological diseases.

#### hAECs in the treatment of stroke

Thus far, only one published study has tested the effect of hAECs on ischemic stroke outcome (Liu et al., 2008). It found that direct intra-cerebral (i.c.) injection of hAECs, 24 h after middle cerebral artery occlusion (MCAO) in rats, resulted in a reduced infarct volume and improved behavioral and neurological outcomes at 16 days post-stroke. Furthermore, apoptosis—as detected via cleaved caspase-3 levels—was reduced in the vicinity of the transplanted cells. It is worth noting that the same research group has also reported that i.c. injection of human amnion mesenchymal cells can similarly improve stroke outcome in rats (Tao et al., 2012). In analogous studies to those in ischemic stroke, intraventricular injection of hAECs was reported to reduce brain edema and to improve motor deficit in a rat model of i.c. hemorrhage (Dong et al., 2010). Moreover, intra-cerebroventricular transplantation of amniotic fluid-derived stem cells at 3 days post-MCAO resulted in the attenuation of stroke-associated cognitive deficits (Rehni et al., 2007).

Despite these very promising early experimental findings, i.c. injection of stem cells still seems unlikely to become a feasible routine method of delivery for stroke patients. Reasons for this include the fact that i.c. injections would require routine access to suitable imaging facilities and surgical expertise, and in any case they may involve significant adverse effects, such as the breakdown of the BBB and a heightened inflammatory response within the brain (McCluskey et al., 2008). Furthermore, any protective effects from i.c. injection of stem cells will presumably be localized to the immediate region of brain and would not also target the detrimental systemic/immunological effects of stroke. Therefore, future studies would ideally employ a less invasive and more clinically amenable delivery route of stem cells, such as intravenous (i.v.) injection.

As i.v. administration is a minimally invasive procedure, it poses a substantially lower risk of adverse clinical events when compared to i.c. transplantation. In fact, Tarjiri and colleagues have reported that i.v. administration of amniotic fluid-derived stem cells at 35 days post-stroke significantly reduces infarct damage and behavioral deficits as assessed at 60–63 days after MCAO (Tajiri et al., 2012). There is therefore great scope to further explore the ability of hAECs to limit injury and/or promote tissue repair and functional recovery when administered systemically following stroke.

#### Potential mechanisms of action by hAECs in the treatment of stroke

**Figure 2:**
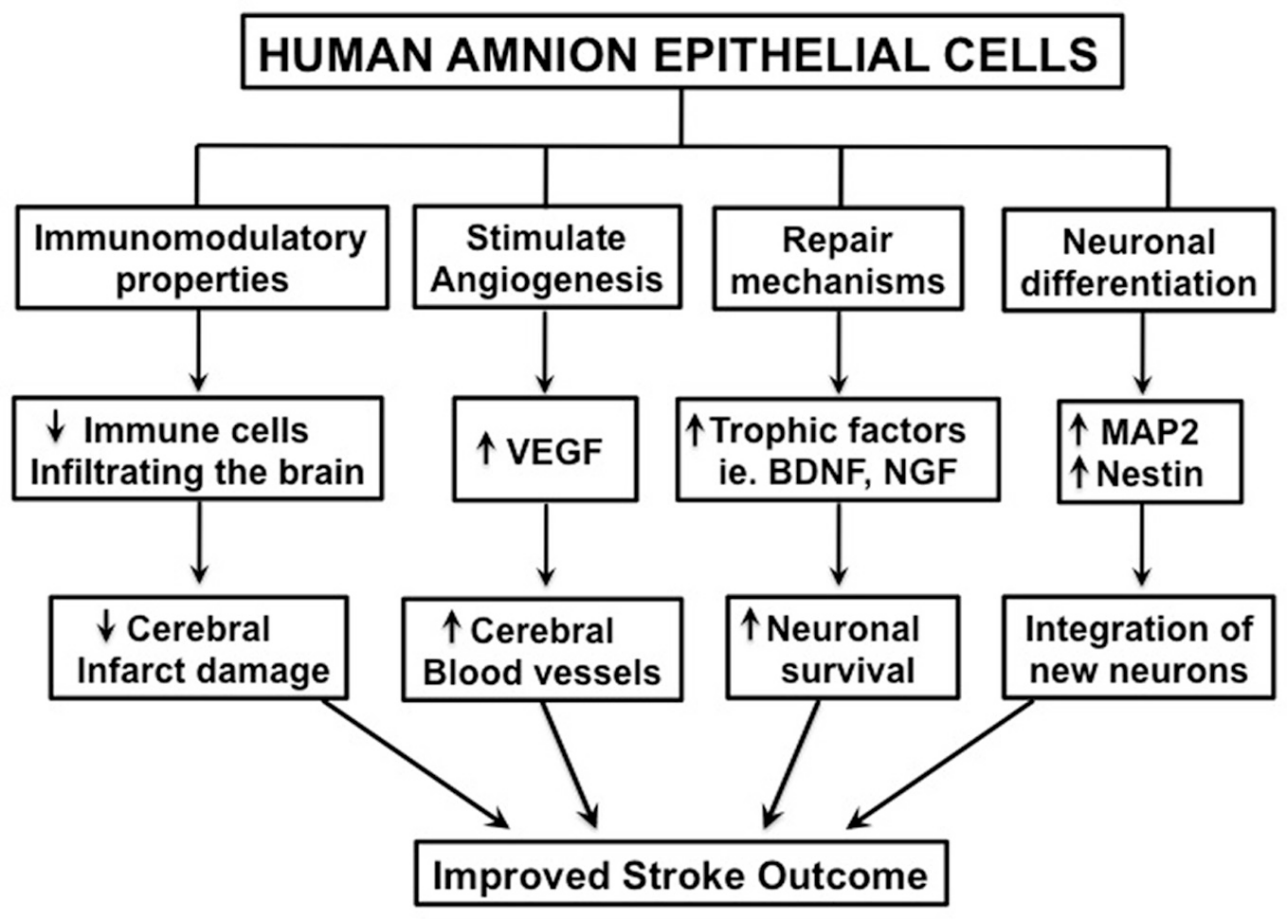
**Schematic diagram illustrating the potential mechanisms by which human amnion epithelial cells may improve stroke outcome.** “↑” = increased; “↓” = decreased.

There are various possible mechanisms by which hAECs might exert therapeutic effects following stroke. Firstly, hAECs could secrete neurotrophic factors that promote neuronal recovery of damaged cells in the penumbra (Lindvall and Kokaia, 2004; Liu et al., 2008). Such factors could also promote synaptogenesis to re-innervate lost connections. Secondly, as hAECs have pluripotent properties, they could differentiate into a neuronal phenotype and replace damaged or dead cells (Lindvall and Kokaia, 2004; Liu et al., 2008). Thirdly, hAECs could act as “biological minipumps” within the CNS, secreting necessary cytokines, growth factors, hormones, and/or neurotransmitters to restore cellular function. Lastly, hAECs could potentially improve stroke outcome by modulating the inflammatory response that contributes to brain injury (Lindvall and Kokaia, 2004; Meisel and Meisel, 2011). Included in this mechanism is the protection of neurons from immune cell-mediated apoptosis (see Figure 2).

#### Immunomodulatory properties of hAECs

hAECs can exert immunomodulatory actions by actively suppressing T lymphocyte proliferation, reducing the expression of the potent pro-inflammatory cytokines IL-1α and IL-1β (Solomon et al., 2001), and via producing inhibitors of MMPs and proteolytic enzymes associated with inflammatory reactions. In addition, although expression of HLA-G on hAECs enables their evasion of the immune system, this protein has also been shown to be anti-inflammatory by inducing apoptosis of activated CD8^+^ T lymphocytes and inhibiting CD4^+^ T lymphocyte proliferation (Banas et al., 2008). Furthermore, hAECs transplanted to the ocular surface can create a local environment that reduces the surrounding inflammatory response (Hori et al., 2006). This effect is thought to be due to hAECs reducing infiltration of major histocompatibility complex class II antigen-presenting cells into the inflamed cornea. Moreover, we have demonstrated that hAECs transplanted into a bleomycin-induced lung injury model reduces the immune response, preventing lung fibrosis and loss of function (Moodley et al., 2010; Murphy et al., 2011). These results were associated with an *in vivo* reduction in the pro-inflammatory cytokines, TNF, IFNγ and IL-6, and an increase in the anti-inflammatory cytokine, IL-10 (Murphy et al., 2011). As a consequence of these actions of hAECs on the immune system, there is a reduction in the infiltration of immune cells to the area of damage.

hAECs are believed to secrete a number of immunomodulatory factors. In fact, supernatant from hAEC culture can inhibit both innate and adaptive immune cells (Li et al., 2005). For example, hAECs produce alpha-fetoprotein, a protein that reduces immune cell reactivity and suppresses neuroinflammation in a mouse model of multiple sclerosis (Irony-Tur-Sinai et al., 2009). Furthermore, hAECs secrete macrophage inhibitory factor, which inhibits neutrophil and macrophage migration and natural killer cell-mediated cytolysis (Li et al., 2005). Fas ligand and TNF-related apoptosis-inducing ligand are both members of the TNF family that are produced by hAECs, can regulate the immune response through apoptosis of lymphocytes (Li et al., 2005). Moreover, hAECs express transforming growth factor-β, which suppresses immune cell numbers through apoptosis as well (Li et al., 2005). Overall, the immunomodulatory properties of hAECs lead us to speculate that these stem cells may be able to limit the inflammatory response that contributes to infarct formation following stroke.

#### Migration of intravenously injected hAECs after stroke

Due to the acute nature of stroke onset, an i.v. injection is ideal so that therapeutics can be administered quickly after the event. However, i.v. administration of stem cells has two initial obstacles that must be overcome: (1) the ability of the cell to pass through the extensive capillary network of the lungs; and (2) whether the cells can effectively home to stroke-affected regions of tissue in sufficient numbers to provide efficacy. Whether this may occur remains to be tested, but the relatively small diameter of hAECs (8–15 μm) probably increases the likelihood of these cells passing through the lungs, compared with larger stem cell lineages, such MSCs, which do not easily passage across the lungs (Fischer et al., 2009). Indeed, we have reported that only a minor percentage of i.v.-injected hAECs persist in the lungs of control mice, and even in mice in which lung injury has been induced using bleomycin (Moodley et al., 2010). Thus, it is conceivable that i.v.-administered hAECs may have minimal impact on lung function and that a substantial proportion of these cells can pass into the systemic circulation.

Stem cells communicate with each other and their environment via paracrine signaling (Burns et al., 2009). In order to understand why and how cells migrate to their target organs, the relevant chemotactic signal(s) must be identified. While very little is known about the chemotaxis response involved in hAEC migration from the circulation following i.v. transplantation, several studies have defined the mechanisms that attract other types of stem cells to injured sites following stroke. For example, it has been shown that there is an increase in levels of stromal cell-derived factor-1α (SDF-1α) in brains of experimental animal models of stroke (Hill et al., 2004; Robin et al., 2006) and a subsequent decrease in stem cell migration after the addition of an antagonist of the chemokine receptor type 4 (CXCR4) (Robin et al., 2006; Wang et al., 2008). SDF-1α is a growth factor produced by multiple types of mouse and human neural cells, and which functions as a chemokine that is thought to be important for neural progenitor migration during development. It is well-documented that the chemokine interaction between SDF-1α and CXCR4, its cognate receptor commonly expressed on the surface of stem cells, plays a major role in stem cell migration (Robin et al., 2006; Wang et al., 2008). More research is required to clarify whether CXCR4 and/or other factors play a role in hAEC homing and signaling pathways.

#### Ability of hAECs to engraft and differentiate and/or promote neuronal repair

Stem cell therapy was initially considered to be an opportunity for treating stroke patients by ultimately replacing dead neurons with new neurons in the post-stroke infarcted brain. As indicated, hAECs can indeed differentiate toward a neural lineage, which may ultimately add to their potential for post-stroke therapy (Elwan and Sakuragawa, 1997; Bailo et al., 2004; Yu et al., 2009). In fact, i.c. injections of hAECs in rats at 24 h after MCAO were found to migrate to ischemic areas and to then express astrocyte (glial fibrillary acidic protein) and neuronal markers (microtubule-associated protein 2 and nestin) (Liu et al., 2008). Correlating with these observations, the hAEC-treated rats showed improved behavioral and neurological outcomes, as well as reduced infarct damage. Thus, the authors postulated that the functional improvement following hAEC treatment may have been partially due to the newly differentiated neuron-like cells re-establishing connections with surviving host neurons. Similarly, in a hemorrhagic stroke model, hAECs transplanted into the brain were found to express neuron-specific antigens and to improve motor deficits after 4 weeks (Dong et al., 2010). As a further example, i.v. administration of amniotic fluid-derived stem cells resulted in an increased number of cells expressing microtubule-associated protein 2, and the cell proliferation marker, Ki67, in the dentate gyrus and in the subventricular zone of stroked animals, indicating increased neurogenesis (Tajiri et al., 2012). Collectively, the existing evidence supports the concept that hAECs can undergo neural differentiation *in vivo*. Future studies must identify if these newly formed neurons are functional and might be able to integrate within the existing network of cells to substantially replace dead tissue.

As indicated above, secreted paracrine factors may play a key role in hAEC-mediated recovery after stroke. If administered as a delayed post-stroke treatment, it is postulated that hAECs could improve long-term stroke outcome via the release of factors that promote re-innervation, thus restoring synaptic transmitter release to stimulate plastic responses, orchestrating rescue and repair processes, and improving or preserving survival and function of existing neurons. Indeed, it has been shown that hAECs can secrete trophic factors such brain-derived neurotrophic factor, neurotrophin-3, nerve growth factor (Sakuragawa et al., 1996; Meng et al., 2007), and novel epidermal growth-like factors (Venkatachalam et al., 2009). Whilst repair of stroke-induced damaged neurons has yet to be described, hAECs have shown an ability to promote recovery of injured tissue and facilitate functional plasticity in other diseases of the CNS. For example, transplanted hAECs produce neurotrophic substances and stimulate repair and regeneration of host neurons in a primate model of spinal cord injury (Sankar and Muthusamy, 2003). In addition, hAECs transplanted into the cerebral lateral ventricle of a transgenic mouse model of Alzheimer's disease were found to rescue damaged cholinergic neurons (Xue et al., 2012). The authors reported that hAEC treatment increased the number of cholinergic neurons, as well as the level of acetylcholine produced by these cells, which was suggested to be largely responsible for the reversal of cognitive decline in this animal model. Thus, in such a manner, hAECs may possess an ability to both repair and replace lost neuronal tissue and, together with their other anti-inflammatory characteristics, they may represent a promising cell-based clinical therapy for neurodegenerative diseases, including stroke.

## Conclusions

In summary, hAECs appear to have several advantages over other stem cell lineages as a cell-based therapy, particularly their non-immunogenic and non-tumorigenic properties. There is now evidence that hAECs can cross the BBB where they can engraft, survive for up to 60 days, differentiate into neurons, reduce inflammation and promote regeneration of damaged CNS tissue in animal models of neurological diseases. We suggest that a future concerted experimental focus to characterise the efficacy of post-stroke hAEC therapy may yield valuable information that could be routinely applied in the clinical setting.

### Conflict of interest statement

The authors declare that the research was conducted in the absence of any commercial or financial relationships that could be construed as a potential conflict of interest.
